# miR-132-3p priming enhances the effects of mesenchymal stromal cell-derived exosomes on ameliorating brain ischemic injury

**DOI:** 10.1186/s13287-020-01761-0

**Published:** 2020-06-29

**Authors:** Qunwen Pan, Xiaoli Kuang, Shuyun Cai, Xiang Wang, Donghui Du, Jinju Wang, Yan Wang, Yanyu Chen, Ji Bihl, Yanfang Chen, Bin Zhao, Xiaotang Ma

**Affiliations:** 1 Guangdong Key Laboratory of Age-Related Cardiac and Cerebral Diseases, Institute of Neurology, Affiliated Hospital of Guangdong Medical University, Zhanjiang, 524001 China; 2 Department of Anesthesiology, Affiliated Hospital of Guangdong Medical University, Zhanjiang, 524001 China; 3 Department of Pharmacology and Toxicology, Boonshoft School of Medicine, Wright State University, Dayton, OH 45435 USA

**Keywords:** Mesenchymal stromal cells, Exosome, miR-132-3p, Ischemia and reperfusion, Apoptosis, ROS production

## Abstract

**Backgrounds/aims:**

Mesenchymal stromal cell-derived exosomes (MSC-EXs) could exert protective effects on recipient cells by transferring the contained microRNAs (miRs), and miR-132-3p is one of angiogenic miRs. However, whether the combination of MSC-EXs and miR-132-3p has better effects in ischemic cerebrovascular disease remains unknown.

**Methods:**

Mouse MSCs transfected with scrambler control or miR-132-3p mimics were used to generate MSC-EXs and miR-132-3p-overexpressed MSC-EXs (MSC-EXs^miR-132-3p^). The effects of EXs on hypoxia/reoxygenation (H/R)-injured ECs in ROS generation, apoptosis, and barrier function were analyzed. The levels of RASA1, Ras, phosphorylations of PI3K, Akt and endothelial nitric oxide synthesis (eNOS), and tight junction proteins (Claudin-5 and ZO-1) were measured. Ras and PI3K inhibitors were used for pathway analysis. In transient middle cerebral artery occlusion (tMCAO) mouse model, the effects of MSC-EXs on the cerebral vascular ROS production and apoptosis, cerebral vascular density (cMVD), Evans blue extravasation, brain water content, neurological deficit score (NDS), and infarct volume were determined.

**Results:**

MSC-EXs could deliver their carried miR-132-3p into target ECs, which functionally downregulated the target protein RASA1, while upregulated the expression of Ras and the downstream PI3K phosphorylation. Compared to MSC-EXs, MSC-EXs^miR-132-3p^ were more effective in decreasing ROS production, apoptosis, and tight junction disruption in H/R-injured ECs. These effects were associated with increased levels of phosphorylated Akt and eNOS, which could be abolished by PI3K inhibitor (LY294002) or Ras inhibitor (NSC 23766). In the tMCAO mouse model, the infusion of MSC-EXs^miR-132-3p^ was more effective than MSC-EXs in reducing cerebral vascular ROS production, BBB dysfunction, and brain injury.

**Conclusion:**

Our results suggest that miR-132-3p promotes the beneficial effects of MSC-EXs on brain ischemic injury through protecting cerebral EC functions.

## Introduction

Ischemic stroke (IS) is one of the leading causes of death worldwide, and so far, less effective strategy is available for the management of IS. It is characterized by endothelial dysfunction and barrier disruption [1]. Oxidative stress has been shown to play critical roles in inducing endothelial cell (EC) dysfunction, inflammation, and apoptosis [2, 3]. Ischemia and reperfusion (I/R) can induce cerebral vascular oxidative stress and apoptosis which contribute to blood-brain barrier (BBB) disruption and led to the occurrence and development of IS [4]. Thus, protection of BBB integrity and EC functions should be important for attenuating cerebral injury at the early stage of IS.

Mesenchymal stromal cells (MSCs), which are self-renewing multipotent stem cells existing in various organs, have shown protective effects on ischemia-induced endothelial dysfunction and vascular damage by differentiating into functional ECs and secreting angiogenic factors [5, 6]. There are evidence demonstrating that MSCs can protect ECs from oxidative stress and apoptosis via paracrine effects [7, 8]. Recent studies suggest that exosomes (EXs) make a pivotal contribution to the paracrine effects of MSCs [9, 10]. EXs, endosomal origin membrane vesicles secreted by a wide range of cell types, are currently recognized as a novel way of stem cell paracrine action and intercellular communication [11]. They can influence the recipient cell functions via transferring molecular cargoes, such as proteins, microRNAs (miRs), and mRNAs. In comparison with stem cells, stem cell-derived EXs have been suggested as new candidates for the treatment of cardiovascular and cerebrovascular diseases because of their advantages, such as more easily passing through the tissue barrier and less risk of rejection response and oncogenesis [12, 13]. MSC-derived EXs (MSC-EXs) could protect the myocardium from I/R damage [12] and increase neurite remodeling in a rat model of IS [13]. Nevertheless, the effects and mechanisms of MSC-EXs on IS are largely unknown.

Of note, miRs of MSC-EXs have been shown to play critical roles in their effects on regulating recipient cell functions. MSC-EXs could promote angiogenesis by delivering pro-angiogenesis miR424 and miR30b into ECs [14]. MSC-EXs with miR modifications are more effective in regulating target cell functions [10, 15, 16]. miR-132-3p, well known as angiogenesis miRs, could promote EC proliferation and migration and contribute to neovascularization in ischemia hindlimb by suppressing the targeted RASA1 expression and activating the Ras signaling pathway [17–19]. The Ras-phosphoinositide 3-kinase (Ras-PI3K) signaling pathway has been found to involve in regulating EC proliferation and apoptosis [20], and activation of the PI3K/Akt/eNOS pathway can decrease oxidized low-density lipoprotein-induced ROS overproduction in ECs [21]. Moreover, a present study has demonstrated that neuronal EXs could regulate tight junction protein Claudin-5 expression and integrity of brain ECs via transferring miR-132-3p [22]. Thus, enrichment of miR-132-3p in MSC-EXs might enhance the effects of MSC-EXs on I/R-induced EC dysfunction.

In the present study, we determined the beneficial effects of miR-132-3p-enriched MSC-EXs on oxidative stress, apoptosis, barrier disruption, and cerebral injury in H/R-induced ECs injury and mouse IS models and investigated the underlying mechanisms via analyzing the Ras/PI3K/Akt/eNOS pathway.

## Materials and methods

### Cell culture

Mesenchymal stem cells (MSCs) were isolated from the bone marrow as previously described [23]. In brief, the bone marrow was collected from the femurs and tibias of 6–8-week-old C57BL/6 mice by flushing with culture medium (DMEM; Gibco, USA). The cells were isolated by using the gradient centrifuge method and resuspended in DMEM with 10% fetal bovine serum (FBS, Gibco). The cells were cultured in DMEM supplemented with 10% FBS in a 37 °C incubator with a humidified atmosphere of 5% CO_2_/95% air. Cells were digested to conventional passage or cryopreservation when the cells grew to 80% confluence.

The microvascular endothelial cells (ECs) of the mouse brain were isolated based on a previous report [24]. Briefly, the microvessels were separated from the cerebral cortices devoid of meninges and big vessels. After being washed with PBS and centrifuge at 1000×*g* for 5 min, the microvessel pellets were resuspended in 10 mL endothelial cell culture medium (10% FBS, 30 μg/mL ECGS, 15 U/mL heparin, 325 μg/mL glutathione, 1 μL/mL 2-mercaptoethanol, 100 U/mL penicillin, and 100 μg/mL streptomycin, Sigma) and plated on rat tail collagen 1-coated 6-well cell culture plates at 37 °C with 5% CO_2_ in the air.

### MSC characterization

The surface marker of MSCs (P3-P6) was identified by flow cytometry, using antibodies against CD44 (BD Biosciences), CD34 (BD Biosciences), CD29 (BD Biosciences), and CD45 (BD Biosciences). For osteogenic differentiation, MSCs from passage 3 were seeded in 6-well plates and cultured with MEM medium (100 nM dexamethasone, 0.05 μM ascorbate-2-phosphate, 10 mM β-glycerophosphate, 100 U/mL penicillin, 100 μg/mL streptomycin, and 10% FBS, Invitrogen), and the cells were incubated in this medium for 3 weeks; osteogenic differentiation was assessed via staining with Alizarin Red (Sigma). The morphology of MSCs was also observed under an inverted microscope (Life Technologies, USA).

### Transfection of MSCs with Lv-miR-132-3p

The lentivirus carrying green fluorescent protein (GFP) marker with murine miR-132-3p (Lv-miR-132-3p) or scrambled control (Lv-SC) were purchased from GenePharma (Shanghai, China). As previously described with some modifications [21], MSCs were transfected with Lv-miR-132-3p or Lv-SC to obtain miR-132-3p-overexpressing MSC and controls, defined as MSC^miR-132-3p^ and MSCs (MSC^SC^). In brief, MSCs were cultured in 6-well plates (1 × 10^5^ cells/well) and incubated with MSC culture medium containing the lentivirus (at 1 × 10^7^ infection-forming units) for 24 h. After that, the medium was refreshed. The positive cells were observed under a fluorescent microscope.

### Preparation and identification of MSC-EXs

After transfection, the MSCs were cultured in 100-mm plates for 24 h, and then the culture medium was collected and centrifuged at 2000*g* for 20 min to remove cells and debris. The collected medium was ultra-centrifuged at 20,000*g* for 90 min and then at 160,000*g* for 3 h to pellet EXs. EXs collected from MSC^SC^ and MSC^miR-132-3p^ were denoted as MSC-EXs and MSC-EXs^miR-132-3p^, respectively. The pelleted MSC-EXs were resuspended with filtered phosphate-buffered saline (PBS) and aliquot for nanoparticle tract analysis (NTA). In addition, the EX-specific markers CD63 and TGS101 were measured by western blot analysis.

### Nanoparticle tracking analysis

The number and size of EXs were determined by the NanoSight NS300 instrument as we previously described [25]. In this study, diluted suspensions containing MSC-EXs were loaded into the sample chamber, and the camera level was maintained at 9 for light scatter mode. The light scatter mode of NTA used the camera filter 1. Three videos of typically 30-s duration were taken, with a frame rate of 30 frames per second. Data was analyzed by the NTA 3.3 software (Malvern Instruments) which was optimized to first identify and then track each particle on a frame-by-frame basis.

### Co-culture assay of MSC-EXs with ECs

MSCs were labeled with PKH26, a red fluorescence cell membrane dye, according to the manufacturer’s protocol with some modifications. Briefly, MSCs were labeled with PKH26 (2 μM) at room temperature (RT) for 5 min. An equal volume of 1% bovine serum albumin (BSA) was added to stop staining. EXs were isolated from the culture medium of PKH26-labeled MSCs. The PKH26-labeled MSC-EXs (50 μg/mL) were added to co-culture (37 °C, 5% CO_2_) with ECs seeded on glass plates for 24 h. Then, cells were washed with PBS and incubated with fluorescein isothiocyanate (FITC)-conjugated anti-beta-actin antibody (Abcam, 1:100) for 1 h at room temperature. The incorporation of MSC-EXs into ECs was examined under a fluorescence microscope (Leica, TCS SP5II, Germany).

### Quantitative real-time PCR

The levels of miR-132-3p in MSCs, MSC-EXs, ECs, and microvessels were measured by quantitative real-time PCR. Total miRs were extracted by using the miRNeasy Mini kit (QIAGEN) according to the manufacturer’s instructions. The miR-132-3p cDNA was synthesized using the Hairpin-itTM miR RT-PCR Quantitation kit (GenePharma, Shanghai, China) under 25 °C for 30 min, 42 °C for 30 min, and 85 °C for 5 min. Real-time PCR was conducted on a RT-PCR system (Bio-Rad). The parameters were 95 °C for 3 min and 40 cycles performed at 95 °C for 12 s and 60 °C for 40 s. PCR primers were as follows: 5-CCAGCATAACAGTCTACAGCCA-3 and 5-TATGGTTGTTCACGACTCCTTCAC-3 for miR-132-3p, and 5-CTCGCT TCGGCAGCACA-3 and 5-AACGCT TCACGAATTTGCGT-3 for U6. The level of miR-132-3p was normalized to U6. The relative quantification of the gene expression was determined using the comparative CT method (2^−ΔΔCt^).

### Cell model of H/R injury

The H/R injury model of ECs was produced as we previously described [26]. Briefly, ECs were grown to 80% confluence in diverse culture dishes and then incubated in a hypoxia incubator (1% O_2_, 5% CO_2_, and 94% N_2_; Thermo Fisher Scientific, USA) for 6 h. After that, the cells were reoxygenated by incubation in a standard cell incubator for 24 h. To generate miR-132-3p knock down ECs, cells were treated with lentiviruses carrying miR-132-3p silencing short hairpin RNA (shRNA) (Lv-SimiR-132-3p) or scramble control. During the reoxygenation time, various groups of ECs were co-cultured with MSC-EXs, MSC-EXs^miR-132-3p^, or culture medium (vehicle). For pathway exploration, cells were pretreated with or without Ras inhibitor (NSC 23766, 100 μΜ; Selleckchem) or PI3K inhibitor (LY294002, 20 μΜ; Selleckchem). All experiments were repeated for three times.

### ROS production analysis

Intracellular ROS level was measured by dihydroethidium (DHE; Beyotime) staining followed with flow cytometric analysis according to the manufacturer’s instruments. After treated with MSC-EXs, MSC-EXs^miR-132-3p^, or culture medium, ECs were incubated with 5 μM DHE solutions for 2 h at 37 °C. Flow cytometry was used to detect the fluorescence intensity of ROS in cells.

### Apoptosis analysis

Cell apoptosis was analyzed by the Annexin V-PE/7-AAD apoptosis detection kit (BD Biosciences) according to the manufacturer’s instructions. Briefly, after co-incubation as described above, ECs were fixed and stained with Annexin V-PE and 7-AAD solution followed by flow cytometry analysis.

### Paracellular permeability analysis

The flux of FITC-conjugated dextran (FITC-dextran, 10 kDa, Sigma) across EC monolayer was used to detect the paracellular permeability as we previously described [27]. Briefly, ECs were seeded on 24-well transwell chambers for 3 days of cultivation to form an endothelial barrier. Then, the cells were treated with H/R and EXs as described above. After 24 h co-culture, the flux of FITC-conjugated dextran (FITC-dextran, 10 kDa, Sigma) across EC monolayer was used to measure the paracellular permeability. The relative fluorescence passed through the chamber was measured by using Enspire Manager (PerkinElmer Company, USA) multimode plate reader. The apparent permeability coefficient (Papp) for FITC-dextran across the cells was calculated by the following equation:

$$ \mathrm{Papp}=\frac{\mathrm{dQ}}{\mathrm{dt}}\times \frac{1}{A\times {C}_0\times 60}\left(\mathrm{cm}/\mathrm{s}\right) $$

where dQ/dt is the amount of FITC transported per minute (ng/min), *A* is the surface area of the filter (cm^2^), *C*_0_ is the initial concentration of FITC (ng/mL), and 60 is the conversion from minutes to seconds.

### Animals

Adult C57BL/6 mice with an age of 6–8 weeks were purchased from the Animal Experiment Center of Guangdong Province (Guangzhou, China) and housed in the Animal Care Facility at the Guangdong Medical University. The mice were maintained in a pathogen-free environment with free access to food and water on a 12-h light/dark cycle before and after surgery. Surgeries were performed under 2.5% isoflurane anesthesia with all efforts were made to minimize pain and distress. All experimental procedures were approved by the Laboratory Animal Care and Use Committees at Guangdong Medical University.

### MSC-EX infusion of mouse IS model

Focal ischemic stroke induced by transient middle cerebral artery occlusion surgery (tMCAO) was carried out in mice as we previously described [28]. Ninety minutes after MCAO, the mice were administrated via the tail vein with PBS (vehicle), MSC-EXs, or MSC-EXs^miR-132-3p^ (1 × 10^10^ particles/100 μL in PBS). The sham-operated mice (Control) underwent the same procedure of tMCAO surgery, except that the monofilament was not inserted. Forty-eight hours after MSC-EX infusion, the mice (*n* = 10/group) were used for various measurements including CBF, NDS, Evans blue extravasation, brain water content, cMVD, infarct volume, cerebral EC apoptosis, and cerebral EC ROS production. We measured CBF with laser Doppler flowmetry systems in 10 mice, and then the mice were sacrificed and the brain tissues were used for cMVD analysis. We performed a 5-point scale method to evaluate NDS then mice were sacrificed and we detected infarct volume by TTC staining in 10 mice. For BBB function analysis, Evans blue were injected into mice 48 h after MSC-EX infusion, then mice were sacrificed and brain tissues were used for Evans blue extravasation detection in 10 mice. We performed brain water content analysis to analyze brain edema in 10 mice. Forty-eight hours after MSC-EX infusion, we injected DHE into the mice then the mice were sacrificed for performed DHE staining to measuring cerebral EC ROS production in 10 mice. After 48 h of MSC-EX infusion, the 10 mice were sacrificed and the brain tissues were used for cerebral EC apoptosis analysis.

### Detection of MSC-EXs merging with ECs in the peri-infarct area

The MSC-EXs were labeled with PKH26 (Sigma) and resuspended with PBS for infusion [16]. In brief, after 24 h of infusion, the brains were dissected from mice and frozen in liquid nitrogen, and then cut into 20-μm-thick sections. The brain sections were incubated with mouse monoclonal to CD31 primary antibody (1:50; Invitrogen) at 4 °C for the night, and the sections were then incubated with goat anti-mouse IgG H&L (Alexa Fluor® 488) secondary antibody for 1 h. After rinsing with wash solution, the sections were observed under a confocal microscope (Olympus Corporation, Japan) for determining the merge of MSC-EXs with ECs in the peri-infarct area.

### Detection of ROS production in cerebral ECs in the peri-infarct area

ROS production in cerebral ECs was measured using DHE (Beyotime, Molecular Probes) fluoromicrography based on the manufacturer’s instructions. DHE (2 μM) was superfused cortically for 60 min by intracardiac injection. At the end of the perfusion, the mice were sacrificed and the brains were quickly removed and frozen in − 80 °C, cut into 20 μm, and incubated with CD31 for microvessels (1:200, Abcam, USA) at 4 °C overnight, following with incubation of Cy3 goat anti-mouse secondary antibody (1:200, EarthOx, USA) for 60 min. The ROS-dependent vascular fluorescence was observed by confocal microscopy.

### Detection of cerebral EC apoptosis

The cerebral EC apoptosis was detected by the TUNEL assay kit (Beyotime) according to the manufacturer’s instructions. In brief, the mouse brain tissue sections (20 μm) were incubated with CD31 for vascular at 4 °C overnight; after incubated with Cy3 goat anti-mouse secondary antibody, the slices were further incubated with TUNEL working solution for 60 min at 37 °C. The slices were observed under a confocal microscope. The labeled ECs (TUNEL+CD31+) in the peri-infarct area of each section were counted in 6 random fields.

### Measurements of cerebral blood flow and microvascular density

The cerebral blood flow (CBF) of mice from various groups was measured using the PeriCam PSI System (Perimed, Sweden) as we previously described [29]. Briefly, 2 days after the infusion of MSC-EXs, mice were anesthetized and placed on a stereotaxic apparatus. A crossing skin incision was made on the head to expose the whole skull. PeriCam PSI System scanning was performed on the intact skull for approximately 1 min. The relative CBF was calculated using the following formula: CBF of ipsilateral side/CBF of contralateral side × 100%. After CBF measurement, the mice were sacrificed and fresh brain tissues were used for cMVD analysis. The cMVD was measured as we previously described by using CD31 (1:50; Invitrogen) staining [28].

### Measurements of infarct volume and neurological deficits

The infarct volume and neurological deficit scores (NDS) were measured by 2% 2,3,5-triphenyltetranzolium chloride (TTC) staining and 5-point scale method as we previously reported [28].

### Evans blue extravasation and brain water content analysis

Evans blue dye was used to evaluate I/R-induced BBB disruption [30]. Briefly, Evans blue dye (4%) in 0.9% saline (2 mL/kg) was injected into the tail vein for 3 h. Then, mice were transcardially perfused with 4% paraformaldehyde (PFA) buffer and sacrificed. The mouse brains were sliced into four 2-mm-thick coronal sections. Furthermore, the ischemic hemispheres were homogenized in 1 mL of 50% triloroacetic acid and centrifuged. The amount of extravasated Evans blue was expressed as nanograms per ischemic hemisphere.

Brain water content in the ischemic hemisphere of mouse brains was measured as described [30]. In brief, the mice were sacrificed, and the brains were separated and weighted (wet weight), followed by drying in an oven at 120 °C for 48 h and weighted again (dry weight). The brain water content was calculated as follows:

(Wet weight − dry weight)/wet weight × 100%

### Immunofluorescence assay

ECs and brain coronal sections were incubated with Ras (1:500; Abcam), Claudin-5, (1:100, Invitrogen), ZO-1 (1:100; Invitrogen), and CD31 (1:50; Invitrogen) antibody overnight at 4 °C. Then, the cells and brain sections were incubated with FITC (green, for Claudin-5 and ZO-1) or Cy3 (red, for CD31) conjugated secondary antibodies (1:250; Invitrogen) for 30 min at room temperature in the dark. Next, the cells and brain sections were washed triple using wash buffer (Beyotime, China), and the cells were incubated with dye for F-actin (rhodamine-phalloidin, 1:1000) for 1 h at room temperature. Cellular nuclear was stained with DAPI (1:1000, Abcam) for 7 min at room temperature. After washing with wash buffer for three times, the fluorescence intensity was detected under a confocal microscope (Leica, TCS SP5II, Germany).

### Western blotting analysis

The proteins of ECs were extracted with cell lysis buffer (Applygen Technologies Company, China) supplemented with protease inhibitor tablet (Thermo Scientific, USA). Protein lysates were electrophoresed trough SDS-PAGE gels and transferred onto PVDF membranes. The membranes were blocked with 5% non-fat milk for 1 h and incubated with primary antibodies against β-actin (1:1000, EarthOx, San Francisco, CA, USA), cleaved Caspase-3 (1:1000, CST, USA), Ras (1:500; Abcam), Claudin-5, (1:100, Invitrogen), ZO-1 (1:100; Invitrogen), PI3K and phospho-PI3K (1:1000, Abcam), Akt and phospho-Akt (1:1000, CST, USA), eNOS and phospho-eNOS (1:1000, Abcam, USA), TGS101 (1:400, Abcam), and CD63 (1:400, Abcam). Blots were developed with ECL solution (Amersham, Sweden).

### Statistical analysis

All data are expressed as mean ± SEM. Multiple comparisons were analyzed by one- or two-way ANOVA followed by a least significant distance (LSD) post hoc test. The GraphPad Prism 7 software was used for analyzing the data. For all measurements, a *p* < 0.05 was considered statistically significant.

## Results

### Generation and characterization of MSCs and miR-132-3p-enriched MSC-EXs

**Fig. 1:**
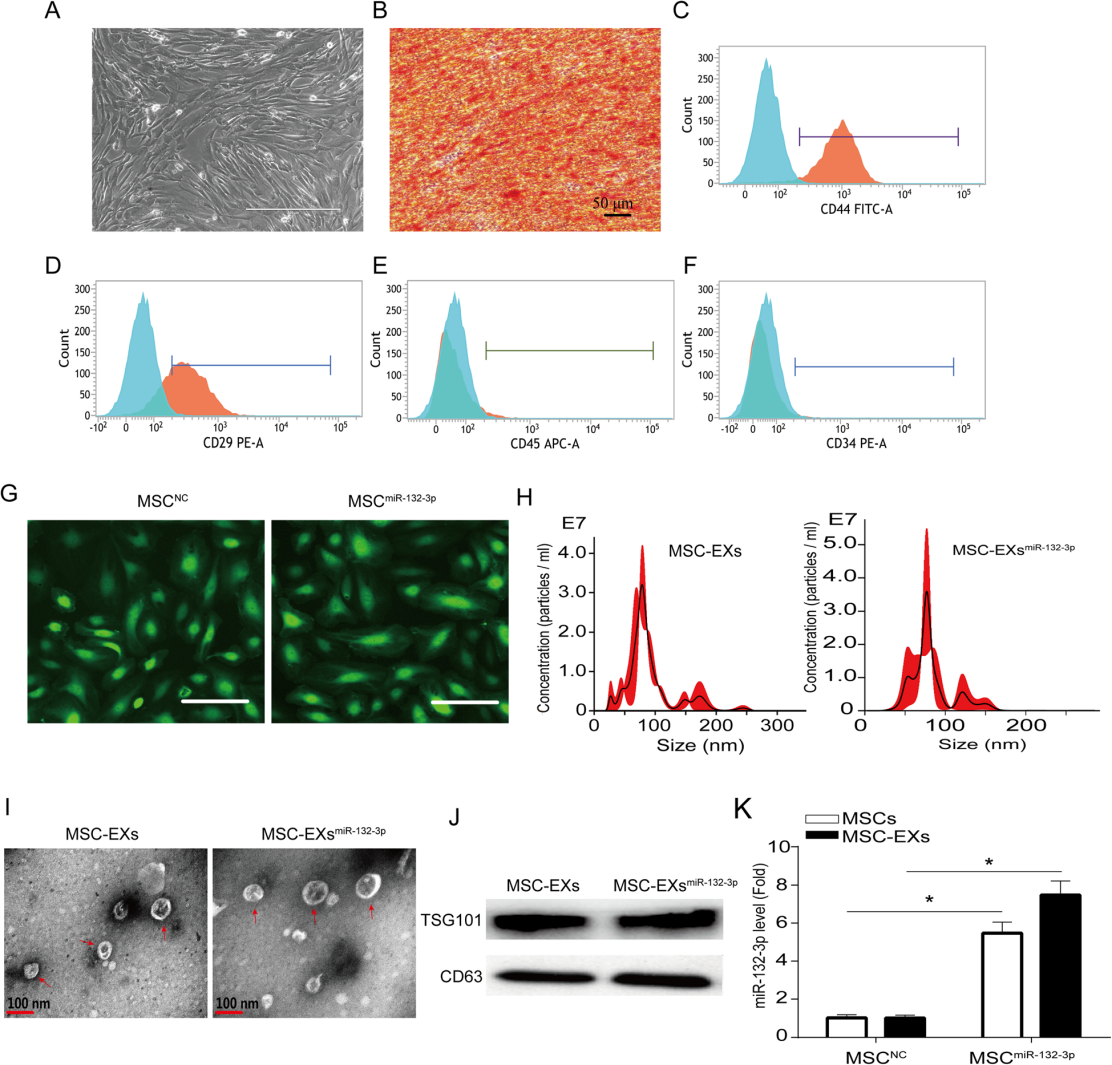
Characterization of MSCs and MSC-EXs. **a** Morphology of MSCs, scale bar = 400 μm. **b** Alizarin Red S staining of osteogenic differentiation of MSCs, scale bar = 50 μm. **c**–**f** Analysis of cell surface antigens of MSCs. **g** Microscopy images of GFP marker expression in MSCs after lentivirus infection, scale bars = 200 μm. **h** The numbers and size of MSC-EXs were detected by NTA. **i** TEM was used to detect the size and morphology of EXs, scale bars = 100 nm. **j** The level of EX-specific marker CD63 and TSG101 was detected by western blot. **k** Real-time PCR results show the level of miR-132-3p in MSCs and MSC-EXs

MSCs typically displayed a characteristic fibroblast-like morphology (Fig. 1a) and with the ability to differentiate into osteoblasts (Fig. 1b). Flow cytometry was used to identify the surface markers of MSCs. The results showed that MSCs were negative for CD34 and CD45 but positive for CD44 and CD29 (Fig. 1c–f). Lenti-miR-132-3p was successfully transfected into MSCs as indicated by the presence of GFP marker (Fig. 1g). The efficiency of miR-132-3p overexpression was evaluated by qRT-PCR analysis. The lenti-NC-transfected MSCs (MSC^NC^) and their derived EXs (MSC-EXs) were set as the controls. We found that the levels of miR-132-3p in MSCs infected with lenti-miR-132-3p (MSC^miR-132-3p^) and their derived EXs (MSC-EXs^miR-132-3p^) were significantly increased (vs MSC^NC^ or MSC-EXs; *p* < 0.05; Fig. 1k). NTA and TEM analyses showed that the size of MSC-EXs was nearby 100 ± 55 nm (Fig. 1h, i). By using western blot analysis, we verified the expressions of special markers (CD63 and TSG101) in isolated MSC-EXs (Fig. 1j). These data indicated that we successfully produced MSC-EXs^miR-132-3p^.

### MSC-EXs modulated the levels of miR-132-3p and RASA1 in ECs compromised by H/R

**Fig. 2:**
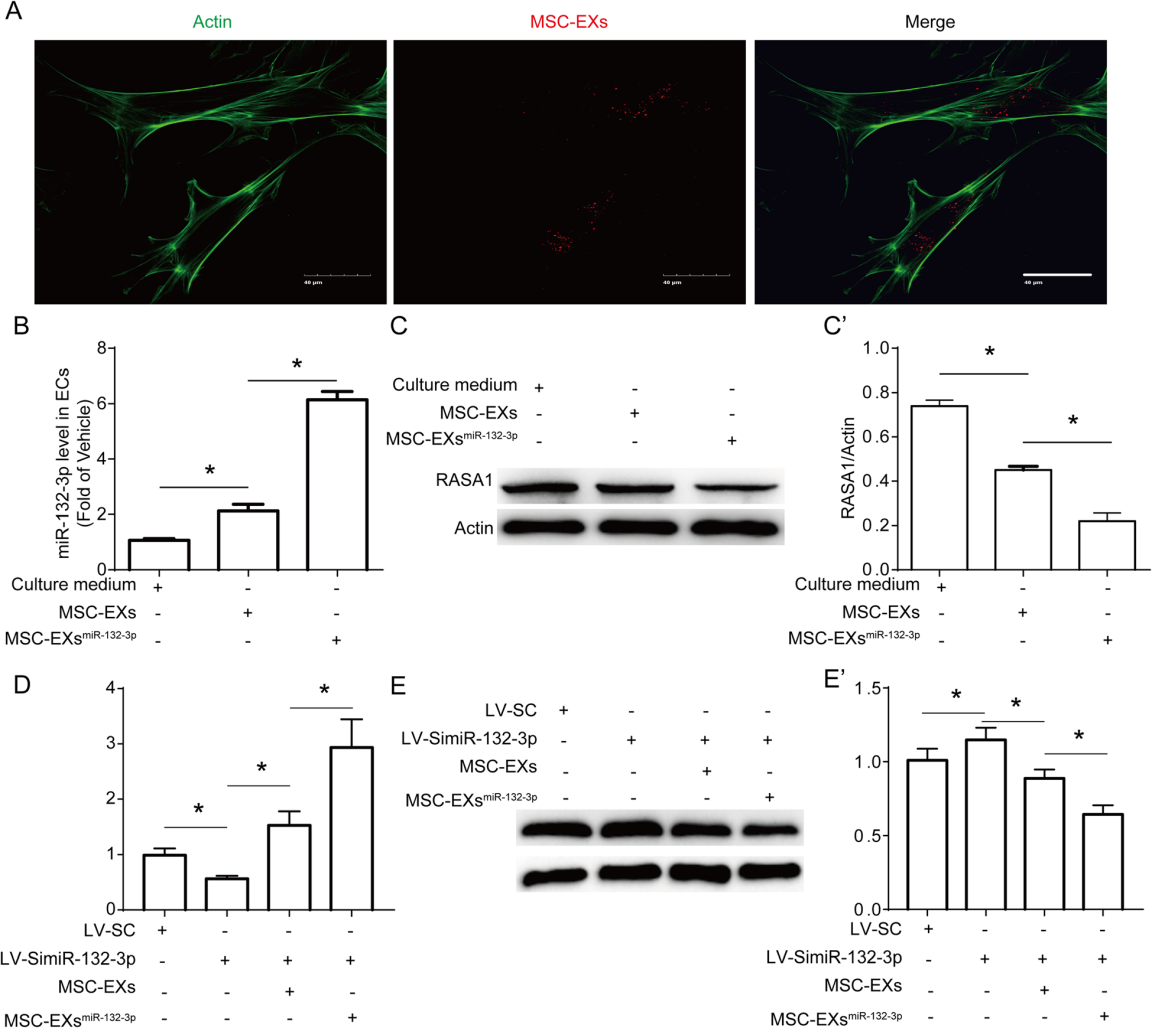
miR-132-3p was delivered from MSCs to ECs by MSC-EXs and decreased its target gene RASA1 expression in ECs. **A** Representative images showing the incorporation of PKH26-labeled MSC-EXs with H/R-injured ECs after 24 h, scale bar = 25 μm. **B** Summarized data of the level of miR-132-3p in ECs. **C**, **C’** Representative bands and summarized data showing the protein level of RASA1 in ECs treated with various MSC-EXs. **D** Summarized data of the level of miR-132-3p in Lv-SimiR-132-3p-treated ECs. **E**, **E’** Representative bands and summarized data showing the protein level of RASA1 in Lv-SimiR-132-3p-treated ECs. *p < 0.05; *n* = 3/group

After co-culture of ECs with PKH26-labeled MSC-EXs for 24 h, we observed MSC-EXs in the cytoplasm of ECs (Fig. 2A), suggesting that MSC-EXs were taken by ECs. To confirm whether MSC-EXs could deliver miR-132-3p to target ECs, qRT-PCR was used to detect the expression of miR-132-3p in ECs or miR-132-3p knock down ECs after co-incubation. As we expected, miR-132-3p expression in MSC-EX-treated ECs was significantly increased (vs vehicle; *p* < 0.05; Fig. 2B) and MSC-EXs^miR-132-3p^ further promoted miR-132-3p expression in ECs (vs vehicle or MSC-EXs; *p* < 0.05; Fig. 2B). Additionally, MSC-EX treatment increased the miR-132-3p expression in Lv-SimiR-132-3p-treated ECs (vs Lv-SimiR-132-3p; *p* < 0.05; Fig. 2D), and MSC-EXsmiR-132-3p were more effective (vs MSC-EXs; *p* < 0.05; Fig. 2D).

Since RASA1 has been proved to be a confirmed target of miR-132-3p [17, 19], we analyzed the regulation of this target in ECs. We found that MSC-EXs decreased the expression of RASA1 in H/R-injured ECs (vs vehicle; *p* < 0.05; Fig. 2C, C’), and MSC-EXs^miR-132-3p^ were more effective (vs vehicle or MSC-EXs; *p* < 0.05; Fig. 2C, C’). In addition, MSC-EXs decreased the RASA1 in H/R-injured ECs with miR-132-3p knock down (vs Lv-SimiR-132-3p; *p* < 0.05; Fig. 2E, E’), and MSC-EXs^miR-132-3p^ treatment was more effective (vs MSC-EXs; *p* < 0.05; Fig. 2E, E’).

### MSC-EXs^miR-132-3p^ were more effective than MSC-EXs on activating the Ras/PI3K/Akt/eNOS signaling in H/R-impaired ECs

**Fig. 3:**
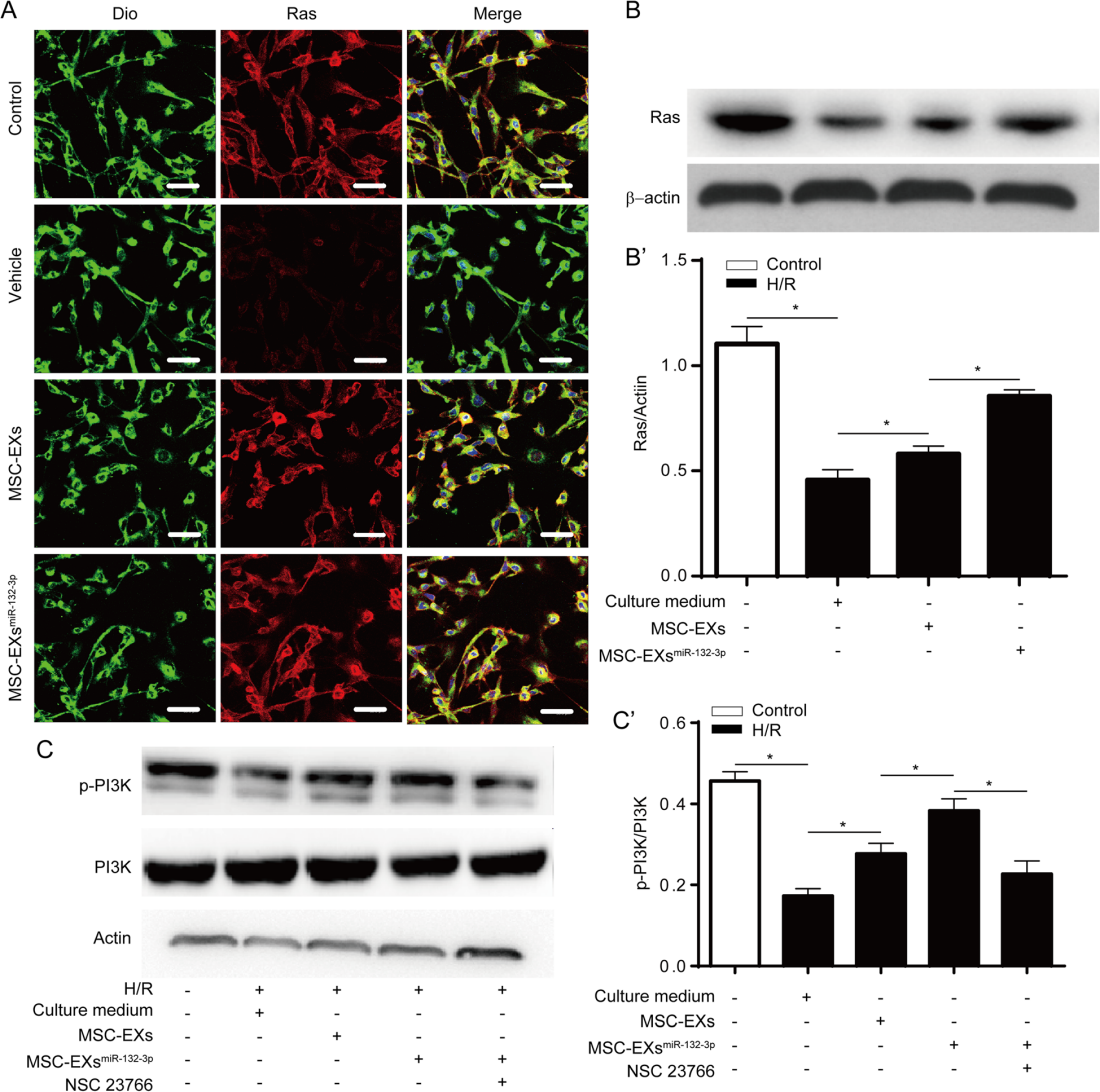
MSC-EXs^miR-132-3p^ were more effective in increasing Ras and p-PI3K/PI3K levels in H/R-injured ECs. **A** The expression and localization of Ras in ECs were detected by immunofluorescence assay, scare bar = 40 μm. **B**, **B’** Representative bands and summarized data showing the protein level of Ras in ECs. **C**, **C’** Representative bands and summarized data showing the protein level of p-PI3K/PI3K. *p < 0.05; *n* = 3/group

We observed that MSC-EXs increased the expression and membrane localization of Ras protein in H/R-injured ECs, and MSC-EXs^miR-132-3p^ had a better efficacy in upregulating the Ras pathway (vs vehicle or MSC-EXs; *p* < 0.05; Fig. 3A, B, B’). Furthermore, we found H/R significantly decreased the phosphorylation level of PI3K in ECs (vs control; *p* < 0.05; Fig. 3C, C’), while MSC-EXs increased the phosphorylation level of PI3K in H/R-injured ECs (vs vehicle; *p* < 0.05; Fig. 3C, C’). Again, MSC-EXs^miR-132-3^were better than MSC-EXs in upregulating the phosphorylation level of PI3K in H/R-injured ECs (vs vehicle or MSC-EXs; *p* < 0.05; Fig 3C, C’). Moreover, these effects of MSC-EXs^miR-132-3p^ were inhibited by Ras inhibitor (vs MSC-EXs^miR-132-3p^; *p* < 0.05; Fig. 3C, C’). The data together indicate that miR-132-3p is a functional content in MSC-EXs in regulating the Ras/PI3K signaling pathway in H/R-injured ECs.

**Fig. 4:**
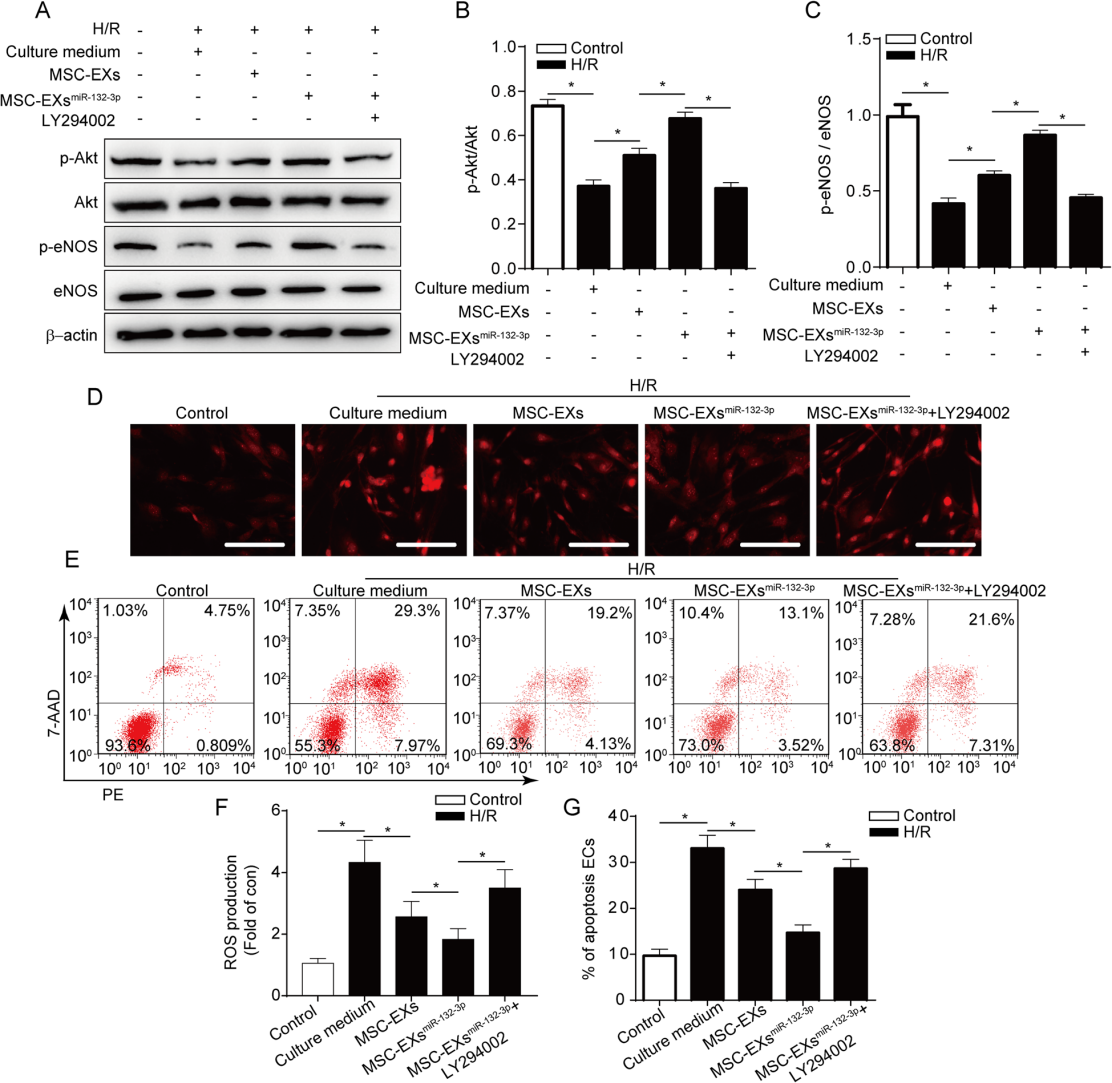
miR-132-3p priming enhanced the effects of MSC-EXs on decreasing ROS production and apoptosis of H/R-injured ECs via activating the PI3K/Akt/eNOS pathway. **a**–**c** Representative bands and summarized data showing the protein level of p-Akt/Akt and p-eNOS/eNOS in ECs. **d**, **f** Representative images and summarized data showing the ROS production of ECs treated by various MSC-EXs and PI3K inhibitor (LY294002), scale bar = 100 μm. **e**, **g** Representative images and summarized data showing the apoptosis of ECs treated by various MSC-EXs and PI3K inhibitor (LY294002). *p < 0.05; *n* = 3/group

We further investigated the expression of downstream Akt, p-Akt, eNOS, and p-eNOS proteins. We found that H/R significantly decreased the level of p-Akt/Akt and p-eNOS/eNOS in ECs (vs control; *p* < 0.05; Fig. 4a–c) and that co-culture with MSC-EXs^miR-132-3p^ for 24 h was more effective in increasing the levels of p-Akt/Akt and p-eNOS/eNOS in H/R-treated ECs (vs vehicle or MSC-EXs; *p* < 0.05; Fig. 4a–c). Moreover, these effects of MSC-EXs^miR-132-3p^ were abolished by a PI3K inhibitor LY294002 (vs MSC-EXs^miR-132-3p^; *p* < 0.05; Fig. 4a–c). Altogether, these data suggested that miR-132-3p enrichment enhances the effects of MSC-EXs on activating the Ras/PI3K/Akt/eNOS signaling pathway in H/R-injured ECs.

### MSC-EXs^miR-132-3p^ were more effective than MSC-EXs on decreasing ROS production and apoptosis in H/R-injured ECs via activating the PI3K/Akt/eNOS pathway

We observed that MSC-EXs significantly decreased ROS overproduction in H/R-injured ECs (vs vehicle; *p* < 0.05; Fig. 4d, f), and MSC-EXs^miR-132-3p^ showed a better efficacy (vs vehicle or MSC-EXs; *p* < 0.05; Fig. 4d, f). Moreover, this effect of MSC-EXs^miR-132-3p^ was partially abolished by LY294002 (vs MSC-EXs^miR-132-3p^; *p* < 0.05; Fig. 4d, f). According to the flow cytometry analysis, MSC-EXs decreased the apoptosis of H/R-injured ECs (vs vehicle; *p* < 0.05; Fig. 4e, g), and MSC-EXs^miR-132-3p^ were more effective (vs vehicle or MSC-EXs; *p* < 0.05; Fig. 4e, g). Moreover, this effect of MSC-EXs^miR-132-3p^ was partially abolished by LY294002 (vs MSC-EXs^miR-132-3p^; *p* < 0.05; Fig. 4e, g).

Taken together, these data indicated that miR-132-3p enrichment enhances the effect of MSC-EXs on reducing ROS production and apoptosis of H/R-injured ECs via activating the PI3K/Akt/eNOS signaling pathway.

### miR-132-3p priming enhanced the effects of MSC-EXs on decreasing the permeability and increasing the ZO-1 and Claudin-5 expression of H/R-injured ECs via activating the PI3K/Akt/eNOS pathway

**Fig. 5:**
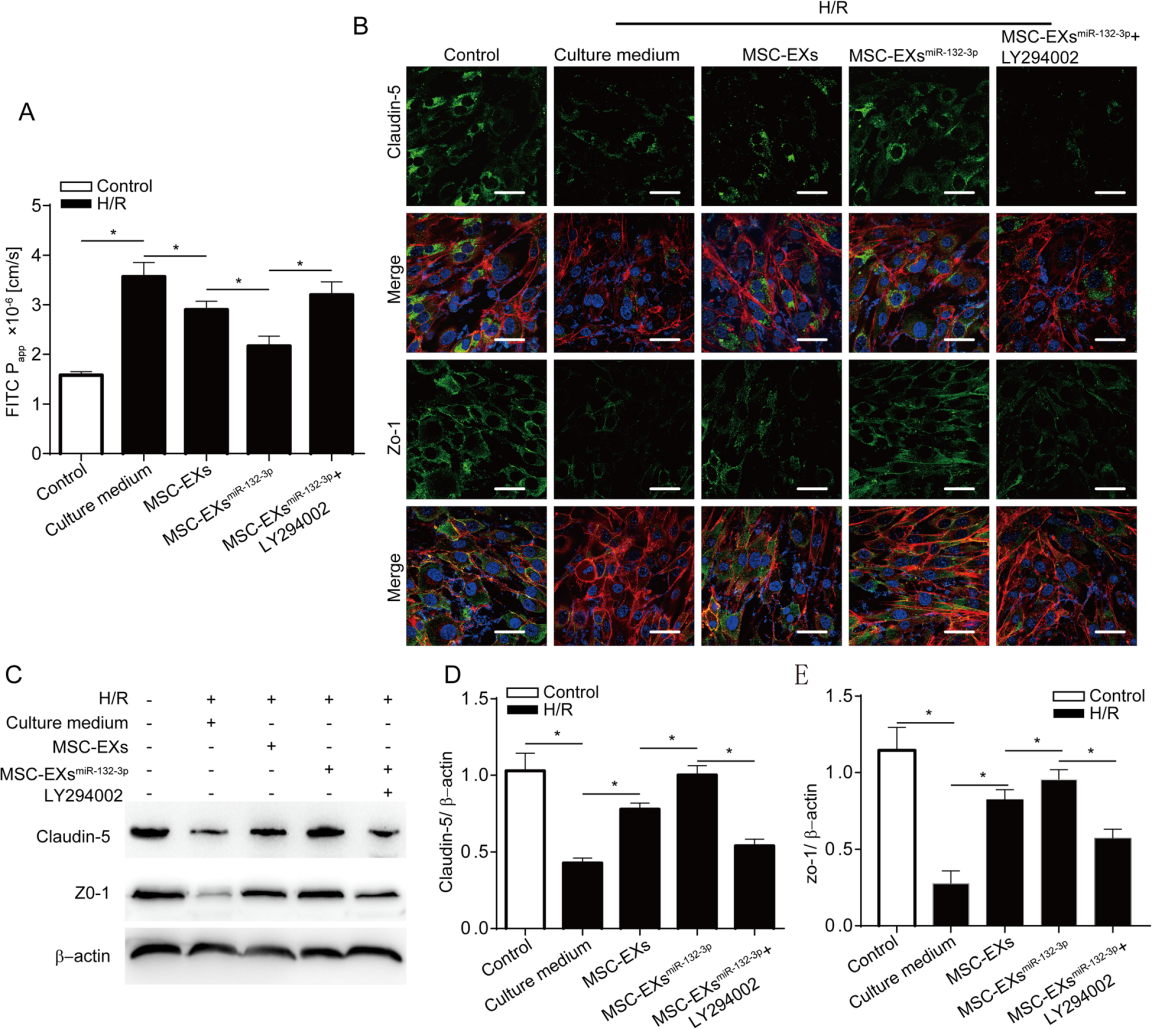
miR-132-3p priming enhanced the effects of MSC-EXs on improving the endothelial tight junction of H/R-injured ECs via activating the PI3K/Akt/eNOS pathway. **a** The permeability of ECs was analyzed by paracellular permeability assay. **b** Representative images of Claudin-5 and ZO-1staining in ECs. **c**–**e** Representative bands and summarized data showing the protein level of Claudin-5 and ZO-1in ECs. *p < 0.05; *n* = 3/group

As shown in Fig. 5, MSC-EXs decreased the paracelluar permeability and increased the expression of ZO-1 and Claudin-5 in H/R-injured ECs (vs vehicle; *p* < 0.05; Fig. 5a–e), and MSC-EXs^miR-132-3p^ had a better efficacy than MSC-EXs (vs vehicle or MSC-EXs; *p* < 0.05; Fig. 5a–e). These effects of MSC-EXs^miR-132-3p^ were partially abolished by a LY294002 (vs MSC-EXs^miR-132-3p^; *p* < 0.05; Fig. 5a–e). These data suggested that miR-132-3p enrichment enhances the effect of MSC-EXs on ameliorating the barrier function via activating the PI3K/Akt/eNOS signaling pathway.

### miR-132-3p priming enhanced the effects of MSC-EXs on decreasing vascular ROS overproduction and apoptosis in tMCAO mice

**Fig. 6:**
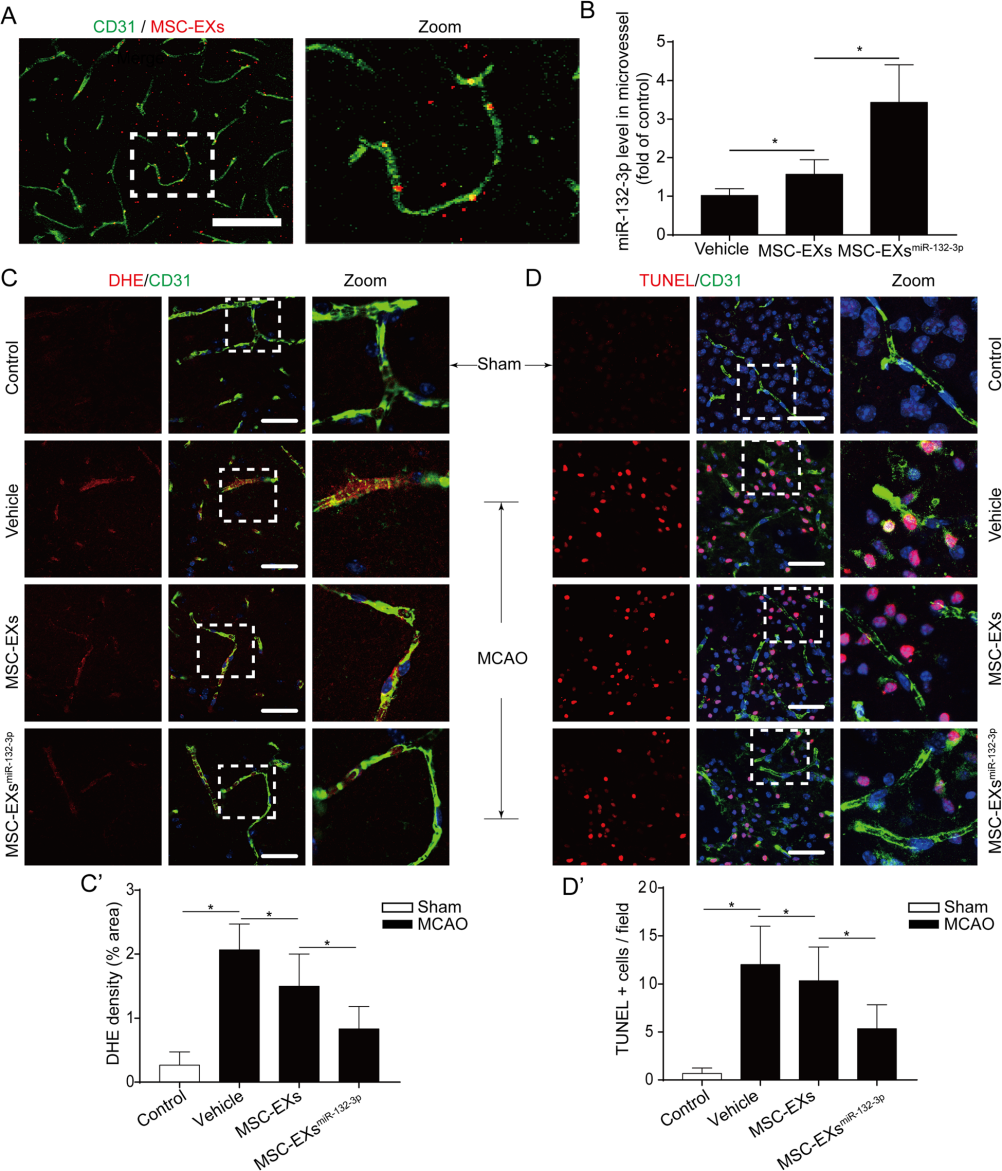
MSC-EXs^miR-132-3p^ were more effective in ameliorating BBB disruption in tMCAO mice. **A** Representative images showing that injected MSC-EXs (PKH26, red) merged with ECs (CD31, green). **B** The level of miR-132-3p in cerebral vascular was detected by qRT-PCR. **C**, **C’** Cerebral vascular ROS production was measured by DHE staining. **D**, **D’** Cerebral vascular apoptosis was measured by TUNEL staining. *p < 0.05; *n* = 10/group

After 24 h of MSC-EX infusion, the fluorescent of PKH26-labeled MSC-EXs was observed in the cerebral microvessels (Fig. 6A). We observed that infusion of MSC-EXs significantly increased the miR-132-3p expression in the cerebral microvessels in the peri-infarct area of tMCAO mice (vs vehicle; *p* < 0.05; Fig. 6B), and infusion of MSC-EXs^miR-132-3p^ is more effective (vs vehicle or MSC-EXs; *p* < 0.05; Fig. 6B).

Based on the anti-oxidative stress and anti-apoptosis effects of MSC-EXs in H/R-injured ECs, we further detected the effects of MSC-EXs on ROS production and apoptosis in the cerebral microvessels in tMCAO mice. We found that infusion of MSC-EXs obviously reduced cerebral vascular ROS overproduction and apoptosis in the peri-infarct area of tMCAO mice (vs vehicle; *p* < 0.05; Fig. 6C–D’), and infusion of MSC-EXs^miR-132-3p^ showed a better efficacy than MSC-EXs in tMCAO mice (vs vehicle or MSC-EXs; *p* < 0.05; Fig. 6C–D’).

Taken together, these data indicated that miR-132-3p enrichment enhances the effects of MSC-EXs on reducing cerebral vascular ROS production and apoptosis in the peri-infarct area of tMCAO mice.

### miR-132-3p priming enhanced the effects of MSC-EXs on alleviating BBB disruption and cerebral injury in tMCAO mice

**Fig. 7:**
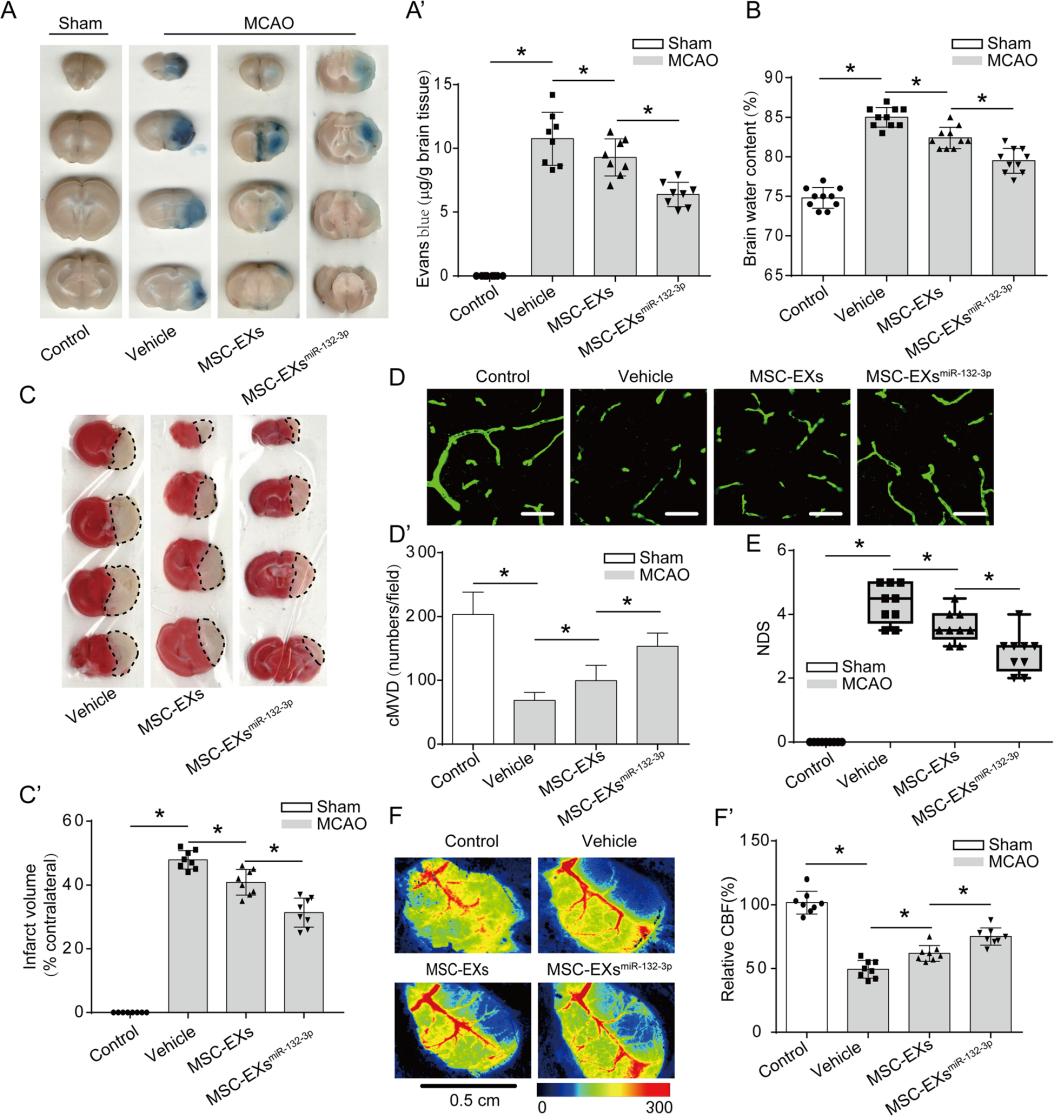
MSC-EXs^miR-132-3p^ was more effective in ameliorating ischemic injury in tMCAO mice. **A**, **A’** The representative images and summarized data of Evans blue extravasation in the ischemic brains of various groups. **B** Summarized data showing the brain water content of IS mice after treated by various MSC-EXs. **C**, **C’** The infarct volume was detected by TTC staining. **D**, **D’** Representative images and summarized data showing the cMVD in the peri-infarct area of MCAO mouse brain. **E** Summarized data showing the NDS of mice. **F**, **F’** Representative images and summarized data showing the CBF in various MSC-EX-treated mice groups. *p < 0.05; *n* = 10/group

The BBB function was detected by analyzing the Evans blue dye extravasation and brain water content in mouse brain tissue. As shown in Fig. 7A–B’, Evans blue dye extravasation and brain water content in MCAO mice were markedly increased at day 2 after tMCAO compared to the sham group (vs control; *p* < 0.05; Fig. 7A–C’). Infusion of MSC-EXs reduced Evans blue dye extravasation and brain water content in tMCAO mice (vs vehicle; *p* < 0.05; Fig. 7A–B’), and MSC-EXs^miR-132-3p^ had a better efficacy on reducing Evans blue dye extravasation and brain water content (vs vehicle or MSC-EXs; *p* < 0.05; Fig. 7A–B’). In cerebral vascular function analysis, we found that infusion of MSC-EXs increased cMVD and CBF in the peri-infarct area of tMCAO mice (vs vehicle; *p* < 0.05; Fig. 7C–D’), and infusion of MSC-EXs^miR-132-3p^ showed a better efficacy on these effects in tMCAO mice (vs vehicle or MSC-EXs; *p* < 0.05; Fig. 7C–D’). Our findings suggest that miR-132-3p enrichment enhanced the effects of MSC-EXs on attenuating BBB disruption and ameliorating cerebral vascular function in tMCAO mice.

The effect of MSC-EXs on cerebral injury was further investigated by measuring the infarct volume and NDS in tMCAO mice. As shown in Fig. 7D–F’, infusion of MSC-EXs significantly decreased the infarct volume and NDS in tMCAO mice (vs vehicle; *p* < 0.05; Fig. 7E–F’), and infusion of MSC-EXs^miR-132-3p^ had a better efficacy on decreasing infarct volume and NDS in tMCAO mice (vs vehicle or MSC-EXs; *p* < 0.05; Fig. 7E–F’). These data indicated that miR-132-3p enrichment enhances the effects of MSC-EXs on alleviating cerebral injury in tMCAO mice.

## Discussion

In the present study, we found that MSC-EXs exerted beneficial effects on protecting ECs from H/R-induced oxidative stress, apoptosis, and tight junction disruption via activating the Ras/PI3K/Akt/eNOS signaling pathway. Loading of miR-132-3p in MSC-EXs boosted these effects. In the mouse tMCAO model, miR-132-3p priming promoted the effects of MSC-EXs on attenuating cerebral vascular and brain injury via decreasing vascular oxidative stress and apoptosis.

Cerebral vascular oxidative stress and apoptosis are the pathological basis for IS [31]. H/R-induced EC injury has been widely used to mimic the in vivo I/R injury. In the present study, we reproduced the model of H/R-injured ECs characterized with increased apoptosis, ROS overproduction, and tight junction disruption. In the cerebral ischemia situation, MSCs can exert vascular protecting effects by directly differentiating into ECs and/or secreting kinds of growth factors [6, 32]. However, the oxidative stress and inflammation environment induced by I/R may impair the differentiation and paracrine effects of MSCs. Accumulating studies have shown multiple benefits for using MSC-EXs rather than MSCs, such as EXs are capable of passing through the blood-brain barrier and they can reduce the potential risks of MSCs therapies, including ectopic tissue formation, unwanted engraftment, infusion toxicities due to cell lodging, and cellular rejection [33, 34]. Therefore, treatment with MSC-EXs may minimize potential adverse effects resulted from ex vivo MSC manipulation. Increasing evidence has demonstrated that EXs could serve as an important and novel way of the paracrine action of stem cells [35, 36]. MSC-EXs could promote tissue repair in myocardial infarction, hind limb ischemia, and IS [37, 38]. In this study, we co-incubated MSC-EXs with H/R-treated ECs and found that H/R-induced ROS overproduction and apoptosis of ECs were markedly decreased by MSC-EXs. Our data indicate that MSC-EXs could promote functional restoration of H/R-injured ECs through reducing cell oxidative stress and apoptosis. As the vascular homeostasis and EC functions are tightly correlated with brain vascular integrity, we further investigated the effects of MSC-EXs on EC barrier function. We found that MSC-EXs increased endothelial barrier function and tight junction protein expression in H/R-injured ECs. In the early stage of I/R injury, EC dysfunction including excess oxidative stress and apoptosis contributes to brain vascular integrity disruption and injury [39]. Thus, we speculate that MSC-EXs could protect cerebral vascular integrity and function from injury by reducing vascular oxidative stress and apoptosis at the early stage of IS.

Nowadays, various methods have been used to isolate EXs from cell culture medium or body fluids, such as ultracentrifugation, density gradient centrifugation, size-exclusion chromatography, precipitation with chemicals, and immunoprecipitation [40]. Ultracentrifugation is a conventional method which uses centrifugal force to separate contaminants from samples containing EVs (apoptotic bodies, microvesicles, and exosomes). To pellet apoptotic bodies, cell culture media or body fluids were centrifuged at 2000*g* at 4 °C, and next, the remaining supernatant can be centrifuged at 20,000*g* to pellet microvesicles (MVs) [25, 41]. Lastly, the remaining supernatant is centrifuged at 100,000*g* to pellet EXs [42, 43]. The major advantages of this method are the low processing cost, the ability to work with large quantities of solution and isolate a large quantity of EXs at once, and the absence of additional chemicals [44]. Therefore, ultracentrifugation has been widely used to isolate EXs from cell culture medium in others [32, 45, 46] and our [42, 47] researches, and the obtained EXs were in the size of nearby 100 nm by NTA and TEM detection and expressed EX-specific marker CD63 and TSG101. Meanwhile, there are some disadvantages to ultracentrifugation, such as the need for ultracentrifugation equipment, the complexity of the stepwise technique, and the efficiency of the technique is dependent on the type of rotor used [48]. Size-exclusion chromatography is another EX isolation method, which makes use of porous beads to separate biomolecules based on their hydrodynamic radius [49]. The purity of preparation, preservation of vesicle integrity, and prevention of EV aggregation are notable advantages for using size-exclusion chromatography [50, 51]. Additionally, the disadvantages include the limitations on sample volume, the need for specialized equipment and a column, and the complexity of the technique [51]. In this study, we selected ultracentrifugation to isolate EXs from the MSC culture medium.

Accumulating evidence indicates that miRs are functional contents in EXs [52, 53]. MSC-EXs are rich in miRs, such as miR-210, miR-126, and miR-130a [14, 54]. As a brain tissue-enriched miR, miR-132-3p plays important roles in promoting EC angiogenesis and maintaining brain vascular integrity [18, 22]. Based on in vitro data that miR-132-3p overexpression restores the high glucose impaired EC proliferation, migration, and tube formation abilities [55] and that miR-132-3p participates in maintaining the integrity of the brain vasculature [22], we overexpressed miR-132-3p in MSC-EXs to study their effects on BBB disruption and cerebral injury. We found that the engineered MSC-EXs containing elevated miR-132-3p could increase miR-132-3p expression in the recipient ECs after co-culture. These data added new evidence to previous studies demonstrating that miR content in the MSC-EXs could be delivered to recipient cells [10, 56]. Our in vitro studies then demonstrated that miR-132-3p further enhanced the effects of MSC-EXs on reducing EC oxidative stress and apoptosis, and endothelial barrier dysfunction in H/R-injured ECs. Previous studies have shown that miR-132-3p is critical for the angiogenic effect of MSC-EXs in ischemic myocardium [57]. This study, for the first time, showed that miR-132-3p promoted the beneficial effects of MSC-EXs on protecting the brain ECs from H/R-induced oxidative stress and apoptosis and then promoted the impaired endothelial barrier function. Nevertheless, the overexpression of miR-132-3p in MSCs may induce alteration of the proteins, lipids, and RNAs in the MSCs, as well as their released EXs, which needs further investigation. The present study demonstrates that engineering the EXs cargoes with miR-132-3p can be a candidate option to promote brain EC and barrier function after I/R injury.

Our present study focused on the RASA1 signaling pathway, since it is a confirmed direct target of miR-132-3p [17, 19], and the miR-132-3p/RASA1 axis is implicated in angiogenesis [19], EC proliferation, migration, and tube formation [55]. A previous study has demonstrated that overexpression of miR-132-3p promotes vascularization and tight junctions of cultured ECs via downregulating RASA1 and subsequent activation of the Ras/MAPK signaling pathway [19]. The RASA1/PI3K/Akt signaling pathway has also been found to involve in regulating EC proliferation and apoptosis [20]. In the present study, we found that MSC-EXs primed with miR-132-3p further downregulated RASA1 expression, as well as activated Ras and the phosphorylation of PI3K, Akt, and eNOS. These data indicate that miR-132-3p-enriched MSC-EXs play an important role in regulating the RASA1/Ras/PI3K/Akt/eNOS pathway. At a cellular level, we verified that miR-132-3p-enriched MSC-EXs decreased ROS overproduction, apoptosis, and impaired barrier function of H/R-injured ECs via activating the PI3K/Akt/eNOS pathway. However, the alterations of the other targets of miR-132-3p, such as SPRED1, Spry1, and eef2k, which mediate the regulation of angiogenesis and vascular integrity, should be further investigated [19, 22].

Stem cell-induced angiogenesis and neurological recovery after IS are highly correlated to stem cell-released EXs [58]. Systemic administration of MSC-EXs has been found to improve functional recovery and enhanced neurovascular function in a rat stroke model [13], and the therapeutic effects of MSC-EXs are highly correlated with their miR contents [16]. A previous study has demonstrated that treatment with EXs derived from miR-133b overexpression MSCs significantly increases neural plasticity and functional recovery after stroke in rats [59]. Based on our in vitro data that miR-132-3p-overexpressing MSC-EXs are more effective in decreasing ROS overproduction and apoptosis, and increasing tight junction of H/R-injured brain ECs, and another report that miR-132-3p-enriched neuronal EXs promoted vascular integrity [22], we carried out our in vivo study by using miR-132-3p-loaded EXs to study their effects on cerebral EC oxidative stress, apoptosis, vascular integrity, and cerebral injury in IS mouse. We found that miR-132-3p-enriched MSC-EXs are more effective in increasing miR-132-3p expression in the cerebral microvessels in comparison with MSC-EXs. As expected, miR-132-3p-enriched MSC-EXs have better efficiency than MSC-EXs on protecting the cerebral microvessels from I/R-induced oxidative stress, apoptosis, and BBB disruption in the peri-infarct area. It is well known that protection of the peri-infarct areas is critical for alleviating IS-induced cerebral injury. In further study, we observed that MSC-EXs have neurovascular protection effects in the acute stage of IS as evidenced by a decrease of infarct volume and NDS, and preservation of cMVD and CBF in the peri-infarct area. Meanwhile, infusion of MSC-EXs^miR-132-3p^ was more effective than MSC-EXs, indicating the enhanced effects on neurovascular protection in the acute stage of IS. However, the distribution, localization, and half-life of administered EXs were not investigated in this study. Further studies are needed to verify their therapeutic impact on the chronic stage of IS.

## Conclusions

In conclusion, our findings suggest that MSC-EXs can be internalized into injured ECs for delivering miR-132-3p in vitro and in vivo and that miR-132-3p enrichment enhances the effects of MSC-EXs on ameliorating H/R-induced endothelial apoptosis and oxidative stress through activating the PI3K/Akt/eNOS pathway and directly repressing RASA1 expression, and on protecting tMCAO-induced ischemic injury via increasing the brain vascular integrity.

## Data Availability

All data generated or analyzed during this study are included in this published article.

## Abbreviations

miR-132-3p: MicroRNA-132-3p
MSC-EXs: Mesenchymal stromal cell-derived exosomes
EXs: Exosomes
H/R: Hypoxia/reoxygenation
ECs: Endothelial cells
p-PI3K: Phosphorylation of phosphoinositide 3-kinase
p-Akt: Phosphorylation-Akt
p-eNOS: Phosphorylation of endothelial nitric oxide synthesis
cMVD: Cerebral vascular density
NDS: Neurological deficit score
I/R: Ischemia and reperfusion
CBF: Cerebral blood flow
BBB: Blood-brain barrier
NTA: Nanoparticle tract analysis
BSA: Bovine serum albumin
MCAO: Middle cerebral artery occlusion
